# Effects of Dietary Supplementation With *Enterococcus faecium* and *Clostridium butyricum*, Either Alone or in Combination, on Growth and Fecal Microbiota Composition of Post-weaning Pigs at a Commercial Farm

**DOI:** 10.3389/fvets.2019.00026

**Published:** 2019-02-28

**Authors:** Yoshiaki Sato, Yasutoshi Kuroki, Kentaro Oka, Motomichi Takahashi, Shengbin Rao, Shin Sukegawa, Tatsuya Fujimura

**Affiliations:** ^1^R&D Center, NH Foods Ltd., Tsukuba, Japan; ^2^Tokyo R&D Center, Miyarisan Pharmaceutical Co., Ltd., Tokyo, Japan

**Keywords:** body weight, *Clostridium butyricum*, *Enterococcus faecium*, gut microbiota, probiotics, swine

## Abstract

Lactic acid bacteria (LAB) and butyric acid bacteria (BAB) are commonly used as probiotics in swine production. However, their combined effect on post-weaning pigs has not been assessed. Therefore, here we investigated the individual and combined efficacy of dietary *Enterococcus faecium* and *Clostridium butyricum* on the growth and gut microbiota of post-weaning pigs at a commercial farm. Four independent trials were conducted, in each of which five pens containing 10 pigs were assigned to one of five treatments: C, basal diet; L, basal diet + live *E. faecium*; D, basal diet + heat-killed *E. faecium*; M, basal diet + *C. butyricum*; or L+M, basal diet + live *E. faecium* + *C. butyricum*. Each trial was conducted over a 90-day period that was divided into two phases (Phase 1, days 0–40 post-weaning; and Phase 2, days 40–90 post-weaning), with the probiotics being supplemented only during Phase 1. Ten pigs in each pen were used for body weight (BW) analysis and fecal samples were collected from five or six of these pigs. In addition, the fecal samples from one randomly selected trial were used for gut microbiota analysis. We found that pigs in the L, D, and L+M treatment groups had a significantly higher BW than those in C (*p* < 0.05) but pigs in the L+M treatment group had a similar BW to those in the L and M groups. Furthermore, there were no significant differences in alpha diversity among the treatments but the beta diversity (weighted UniFrac distances) showed distinct clustering patterns, with pigs in C having discrete microbiota from those in all of the probiotics treatment groups except D (C vs. L, *q* = 0.04; C vs. M, *q* = 0.06; C vs. L+M, *q* = 0.06). These findings indicate that dietary supplementation with live or heat-killed *E. faecium* enhances growth performance in pigs but there is no synergistic effect when *E. faecium* is used in combination with *C. butyricum*. Furthermore, the addition of live *E. faecium* and *C. butyricum* to the diet of pigs may change the structure of the gut microbiota.

## Introduction

Antibiotics are often added to the diets of pigs to reduce the occurrence of diseases such as diarrhea and to improve growth performance. However, there is growing concern about the risk of the emergence of antibiotic-resistant bacteria and the residual effects of antibiotics in meat products. Subsequently, the subtherapeutic use of antibiotics as growth promoters has been banned in Europe since 2006 (Council Regulation EC 70/524/ EEC). Therefore, there has been increased interest in finding a suitable alternative to antibiotics, with probiotics receiving considerable attention.

Lactic acid bacteria (LAB), such as *Lactobacillus, Bacillus*, and *Enterococcus*, are among the most commonly used probiotics for pigs. Many studies have shown that the use of *Enterococcus faecium* has a positive effect on the health and performance of pigs. For example, Mallo et al. (1) reported that dietary supplementation with 10^6^ CFU/g *E. faecium* improved the gut microbiota balance, growth, and feed conversion of piglets, and Zhao et al. (2) demonstrated that the use of *E. faecium* improved growth performance in weanling pigs. However, some studies have yielded inconsistent results–for example, Broom et al. (3) showed that supplementation of the post-weaning diet with *E. faecium* did not affect growth performance or gastrointestinal bacterial populations in piglets. Therefore, it is likely that the effect of dietary *E. faecium* in pigs depends on the diet composition, diversity of bacterial species and strains, as well as environmental factors.

Butyric acid bacteria (BAB), such as *Clostridium*, have also been considered as possible candidates for use as probiotics in swine production. *Clostridium butyricum* is a butyric acid-producing, Gram-positive anaerobe found in soil and in the intestines of healthy animals and humans (4) that has been shown to have beneficial effects on animal health and performance, and it is commonly used as a feed additive in Asia and Europe. Furthermore, Takahashi et al. (5) recently reported that the use of *C. butyricum* may improve the zootechnical performance of weaned piglets.

*Enterococcus faecium* and *C. butyricum* have been widely used for animal production in Japan. *Enterococcus* is facultative anaerobic bacteria, while *Clostridium* is obligate anaerobe bacteria. Due to the characteristics, the two bacteria grow within different sites of the gut. *Enterococcus* can form biofilm microcolonies throughout the gastrointestinal tract (6), but *Clostridium* mainly grow in the distal intestine, cecum and colon (7). Due to their differing characteristics, the two bacteria do not compete for nutrients as they have different ecological niches and it is expected that the combined use of these probiotics would have highly synergistic effects. However, there have been no reports on the combined effects of LAB and BAB on post-weaning pigs. Therefore, the aim of this study was to investigate the efficacy of dietary *E. faecium* and *C. butyricum*, both separately and in combination, on the growth and gut microbiota of post-weaning pigs at a commercial farm.

## Materials and Methods

### Probiotics

Four probiotic powder products (Products 1–4) containing live or heat-killed *E. faecium* strain NHRD IHARA (EFNH), or live *C. butyricum* strain MIYAIRI 588 (CBM588) were manufactured by a commercial company (Miyarisan Pharmaceutical Co., Ltd., Tokyo, Japan). The concentrations of probiotics in the products are shown in Table 1.

**Table 1:** Concentrations of *Enterococcus faecium* and *Clostridium butyricum* in the probiotic powder products.

**Probiotic**	**Product 1**	**Product 2**	**Product 3**	**Product 4**
Live *E. faecium* NHRD IHARA (cfu/g)	1.2 × 10^9^	–	–	1.2 × 10^9^
Heat-killed *E. faecium* NHRD IHARA (cells/g)	–	1.1 × 10^9^	–	–
Live *C. butyricum* MIYAIRI 588 (cfu/g)	–	–	9.6 × 10^7^	2.0 × 10^8^

### Study Design

This study was conducted at a commercial farm in Japan. A total of 200 pigs (Landrace × Large White × Duroc) with initial age of 30.0 ± 1.0 days old were used in the experiment, which involved four independent trials. All trials were started at the first days after weaning of the pigs. In each trial, 50 pigs were housed in five pens at a density of 10 pigs per pen and each pen was randomly assigned to one of five treatment groups: C, basal diet without probiotics; L, basal diet supplemented with 1 g/kg of Product 1 containing live *E. faecium*; D, basal diet supplemented with 1 g/kg of Product 2 containing heat-killed *E. faecium*; M, basal diet supplemented with 1 g/kg of Product 3 containing live *C. butyricum*; and L+M, basal diet supplemented with 1 g/kg of Product 4 containing live *E. faecium* + *C. butyricum*. Each trial was conducted over a 90-day period that was divided into two phases (Phase 1, days 0–40 post-weaning; and Phase 2, days 40–90 post-weaning), with the probiotics being supplemented only during Phase 1. Phase 2 was designed to investigate whether effects of the probiotics were sustained or not. The pigs were fed diets A or B, which were for growing pigs and for adult pigs, respectively (Table 2), and as a basal diet during Phases 1 and 2, respectively. These diets and water were offered *ad libitum*. The experiments were exempted from ethic evaluations because all animals were commercially raised and limited to body weight and microbiological evaluations.

**Table 2:** Chemical compositions (%) of diets A and B.

	**Diet A**	**Diet B**
Crude protein	18.0	14.0
Ether extract	3.0	2.5
Crude fiber	5.0	4.5
Crude ash	8.0	7.5
Calcium	0.55	0.55
Phosphorus	0.40	0.45
Total digestible nutrients	80.0	78.0

### Sample Collection

In each trial, 10 pigs in each pen were used for body weight (BW) analysis. The BW of each animal was measured on the first day of Phase 1 (T1) and on the final days of Phases 1 and 2 (T2, and T3, respectively). Since the weighed pigs were not of the same age, the BW on T2 and T3 were estimated from the measured values by linear approximation, as previously described (8). In addition, fecal samples were collected from five or six of the weighed pigs in each pen on T1, T2, and T3, lyophilized, and stored at −20°C until further analysis.

### DNA Extraction

The fecal samples from one randomly selected trial were used to analyze the gut microbiota in each treatment group. This included 30 samples on T1 (*n* = 6 for each treatment group) and 29 samples on T2 and T3 (*n* = 5 for C; *n* = 6 for all other groups). Bacterial DNA was extracted from the lyophilized feces following a previously described procedure (9, 10) with modification (8). Briefly, fecal samples (20 mg) were washed three times in Tris-EDTA (ethylenediaminetetraacetic acid) buffer (10 mmol/L Tris-HCl, 1 mmol/L EDTA; pH 8.0). Subsequently, the samples were resuspended in a solution containing 500 μL of Tris-EDTA buffer and 100 μL of 10% sodium dodecyl sulfate. Glass beads (300 mg; diameter, 0.1 mm) and 600 μL of buffer-saturated phenol were added. Then the mixture was vortexed for 10 s using Micro Smash MS-100R (TOMY SEICO CO., LTD, Tokyo, Japan) followed by incubation at 65°C for 10 min. This step was repeated twice. After the step, 550 μL of the supernatant was subjected to isopropanol precipitation. The extracted DNA was suspended in 200 μL of Tris-EDTA buffer. Subsequently the DNA was purified using a High Pure PCR Template Kit (Roche Inc., Basel, Switzerland) according to the manufacture's instruction. Finally the DNA was suspended in 50 μL elution buffer and stored at −20°C until further analysis.

### 16S rRNA Gene Amplification and High-Throughput Sequencing

The extracted DNA was amplified using the specific primer pair 341F/805R [341F: 5′-CCTACGGGNGGCWGCAG-3′; 805R: 5′-GACTACHVGGGTATCTAATCC-3′; (11)], which targets the V3–V4 region of the 16S rRNA bacterial gene. This primer set contained the Illumina MiSeq sequencing adapter (forward primer: AATGATACGGCGACCACCGAGATCTACAC; reverse primer: CAAGCAGAAGACGGCATACGAGAT) and a unique barcode sequence that allowed all of the samples to be pooled for Illumina MiSeq sequencing. Each 50 μL of polymerase chain reaction (PCR) mixture contained 1 μL of sample DNA, 21 μL of MilliQ® water, 25 μL of 2X MightyAmp® Buffer, 1 μL of forward primer (5 μM), 1 μL of reverse primer (5 μM), and 1 μL (1.25 U) of MightyAmp DNA Polymerase (Takara, Tokyo, Japan). The following thermal cycling conditions were used: initial denaturation at 98°C for 2 min, followed by 35 cycles at 95°C for 10 s, 60°C for 15 s, and 68°C for 40 s, and final elongation at 72°C for 5 min. The PCR products were checked on a 2% agarose gel for correct product size formation.

Amplicons of 16S rDNA were purified using SPRI select beads (Beckman-Coulter Inc., USA) with a 0.62 × ratio of beads to sample volume. These libraries were quantified using the Quantus™ Fluorometer (Promega, Tokyo, Japan) and pooled in equal amounts of DNA. The pooled library was then sequenced on an Illumina MiSeq platform (Illumina, San Diego, CA, USA), which generated paired 300-bp reads.

After sequencing, six samples from T1 (C, *n* = 3; L, *n* = 2; L+M, *n* = 1) were excluded from the downstream analyses owing to their low numbers of reads.

Sequence data obtained from the present study have been deposited DDBJ under accession no DRA007951.

### Preprocessing and Analysis

Raw sequence data were analyzed with QIIME 2 version 2018.2 (http://qiime2.org). The data were first demultiplexed and processed using DADA2 (12) for quality filtering and the construction of amplicon sequence variants (ASVs), which are analogous to traditional operational taxonomic units (OTUs). The ASVs were then summarized in feature tables, which were rarefied to a depth of 20,000 ASVs using the qiime feature-table rarefy command to avoid the bias that occurs with increasing depth. Representative sequences were aligned with MAFFT (13) using the qiime alignment command and were used to construct a phylogenetic tree with FastTree2 using the qiime phylogeny command (14). Alpha diversity, which was represented by the Chao1 estimator and the Shannon index, and beta diversity, which was represented by the weighted UniFrac distances, were estimated using the q2-diversity plugin in QIIME 2. Principal coordinates analysis (PCoA) based on weighted UniFrac distances was also performed using QIIME 2 and visualized with Emperor (15). The taxonomy of the sequence variants was assigned using the QIIME 2 q2-feature-classifier plugin against the Greengenes13.8 99% OTUs full-length sequences (16).

### Statistical Analyses

The BW data were analyzed using analysis of variance and analysis of covariance with the generalized linear model procedure in R 3.5.0. Normal distribution was evaluated by Shapiro–Wilk test and the homogeneity of variance was tested by Levene's test. The model for the analysis of BW on T1 included treatment and trial fixed effects, while the models for T2 included treatment and trial fixed effects as well as the BW on T1 as a covariate. For analysis of the BW on T1 and T2, the TukeyHSD method was used for multiple comparisons. For analyzing the BW on T3, Games-Howell's test was used for multiple comparisons because of heteroscedasticity of the data (Levene's test; *p* < 0.05). Differences were considered statistically significant at *p* < 0.05.

Differences in the relative abundances of microbial taxa among the treatment groups were tested using the Kruskal–Wallis test followed by the *post hoc* Mann–Whitney *U*-test in R 3.5.0. The Kruskal–Wallis test for multiple pairwise comparisons was also performed to analyze statistical differences in alpha diversity (Chao1 and Shannon index), while permutational multivariate analysis of variance was used with 999 permutations to evaluate differences in weighted UniFrac distances in QIIME 2. In all tests, the *p*-values were adjusted for multiple testing using the Benjamin–Hochberg procedure (*q*-values) and differences among the means were considered statistically significant at *q* < 0.10.

## Results

### Growth Performance

The total of 9 pigs (*C* = 6, *L* = 3) were sick during Phase 2 or 3, and the pigs were excluded from analyzing body weight. There was no significant difference in the corrected BW of pigs among treatment groups on T1 or T3 (Table 3). On T2, pigs in the L, D, and L+M treatment groups had a significantly higher BW than those in C (*p* < 0.05), but pigs in the L+M treatment group had a similar BW to those in the L and M groups.

**Table 3:** Corrected body weight (kg) of the post-weaning pigs in each treatment group.

**Time**	**C**	**L**	**D**	**M**	**L+M**
T1	10.4 ± 1.61	10.0 ± 1.10	10.2 ± 0.74	10.2 ± 1.43	10.2 ± 1.25
T2	31.6 ± 5.04^a^	33.3 ± 3.49^b^	32.9 ± 3.28^b^	31.9 ± 4.42^ab^	32.9 ± 3.89^b^
T3	72.7 ± 8.66	74.6 ± 6.43	74.2 ± 6.86	73.1 ± 8.40	74.5 ± 5.56

*Values are means ± standard deviations*.

ab *Means with different superscript letters within a column are significantly different (p < 0.05)*.

*T1, First day of Phase 1 (day 0 post-weaning); T2, final day of Phase 1 (day 40 post-weaning); T3, final day of Phase 2 (day 90 post-weaning)*.

*C, Basal diet; L, basal diet + 1 g/kg of Product 1 containing live Enterococcus faecium; D, basal diet + 1 g/kg of Product 2 containing heat-killed E. faecium; M, basal diet + 1 g/kg of Product 3 containing live Clostridium butyricum; L+M, basal diet + 1 g/kg of Product 4 containing live E. faecium + C. butyricum*.

### Alpha Diversity

There were no significant differences in alpha diversity (Chao1 and Shannon index) among the treatment groups on T1, T2, or T3 (Table 4).

**Table 4:** Alpha diversity of the gut microbiota of post-weaning pigs in each treatment group.

	**C**	**L**	**D**	**M**	**L+M**
**T1**					
Chao1	537.82 ± 34.50	399.30 ± 150.02	479.69 ± 78.74	429.35 ± 101.11	351.57 ± 62.52
Shannon	7.24 ± 0.18	6.33 ± 0.67	6.58 ± 0.92	6.70 ± 0.35	5.93 ± 1.30
**T2**					
Chao1	696.43 ± 83.54	637.49 ± 78.95	740.50 ± 159.08	668.73 ± 74.58	649.71 ± 187.31
Shannon	7.51 ± 0.25	7.04 ± 0.35	7.39 ± 0.58	7.25 ± 0.27	7.27 ± 0.70
**T3**					
Chao1	347.83 ± 45.04	423.13 ± 103.97	656.42 ± 214.88	436.53 ± 124.64	463.35 ± 136.83
Shannon	6.40 ± 0.21	6.70 ± 0.52	6.77 ± 0.69	6.32 ± 0.68	6.46 ± 0.48

*Values are means ± standard deviations. T1, First day of Phase 1 (day 0 post-weaning); T2, final day of Phase 1 (day 40 post-weaning); T3, final day of Phase 2 (day 90 post-weaning)*.

*C, Basal diet; L, basal diet + 1 g/kg of Product 1 containing live Enterococcus faecium; D, basal diet + 1 g/kg of Product 2 containing heat-killed E. faecium; M, basal diet + 1 g/kg of Product 3 containing live Clostridium butyricum; L+M, basal diet + 1 g/kg of Product 4 containing live E. faecium + C. butyricum*.

### Beta Diversity

**Figure 1:**
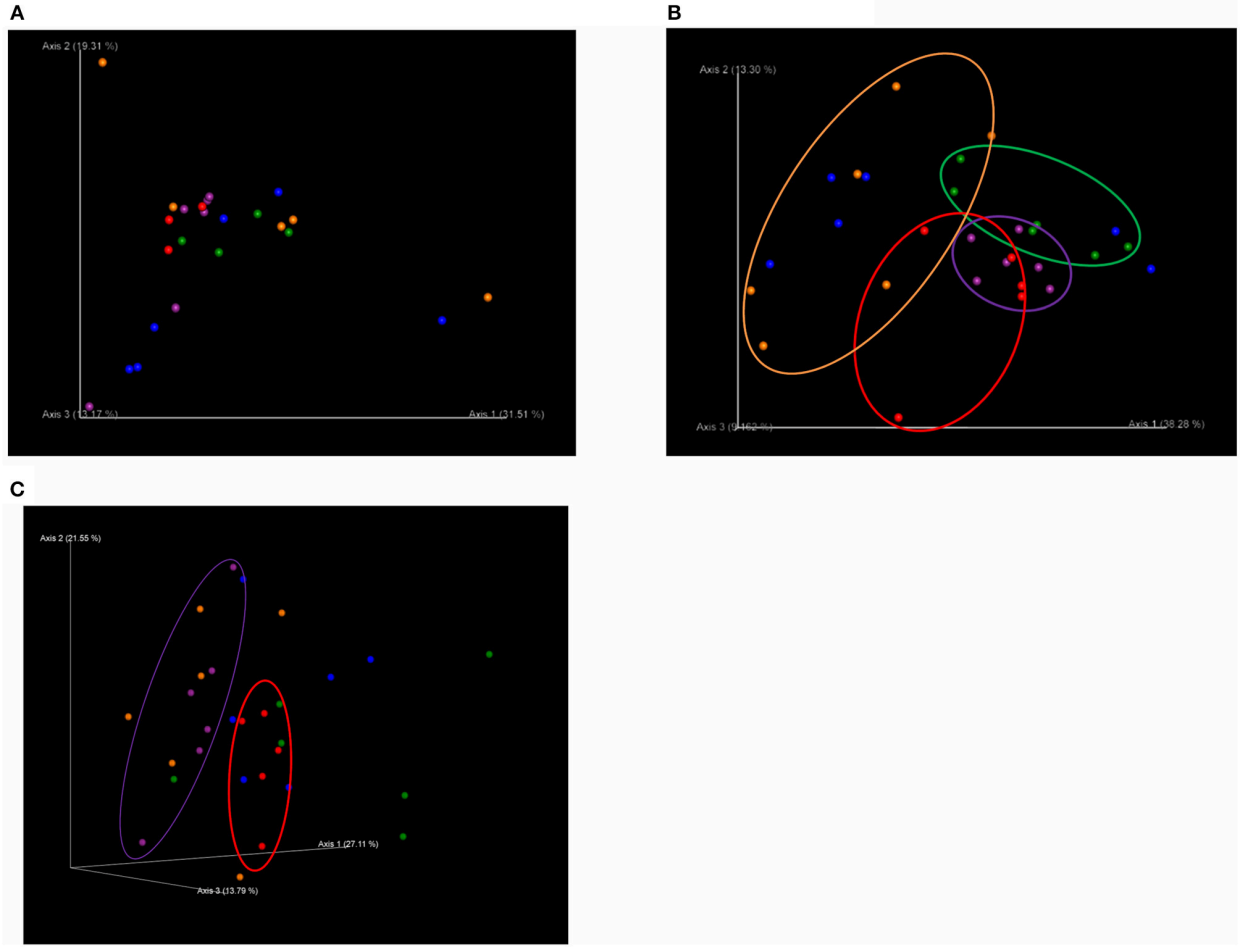
Principal coordinates analysis (PCoA) plots based on weighted UniFrac distances of the bacterial community structures in the gut microbiota of post-weaning pigs that were fed probiotics-supplemented diets or a control diet on **(A)** the first day of Phase 1 (T1; day 0 post-weaning), **(B)** the final day of Phase 1 (T2; day 40 post-weaning), and **(C)** the final day of Phase 2 (T3; day 90 post-weaning). 3D PCoA plots were visualized using the program Emperor. Red: basal diet (C); green: basal diet + 1 g/kg of Product 1 containing live *Enterococcus faecium* (L); blue: basal diet + 1 g/kg of Product 2 containing heat-killed *E. faecium* (D); purple: basal diet + 1 g/kg of Product 3 containing live *Clostridium butyricum* (M); orange: basal diet + 1 g/kg of Product 4 containing live *E. faecium* + *C. butyricum* (L+M).

There was no significant difference in beta diversity (weighted UniFrac distances) among treatments on T1 (Figure 1A). However, on T2, the weighted UniFrac distances showed distinct clustering patterns that separated the microbiota of pigs in C from those in each of the dietary probiotics treatment groups except D (C vs. L, *q* = 0.04; C vs. M, *q* = 0.06; C vs. L+M, *q* = 0.06), as well as pigs in M from those in L+M (*q* = 0.02) and pigs in L from those in M and L+M (*q* = 0.03 for each) (Figure 1B). The microbiota of pigs in M was also different from that of pigs in C on T3 (*q* = 0.08) (Figure 1C).

### Taxonomic Composition

**Figure 2:**
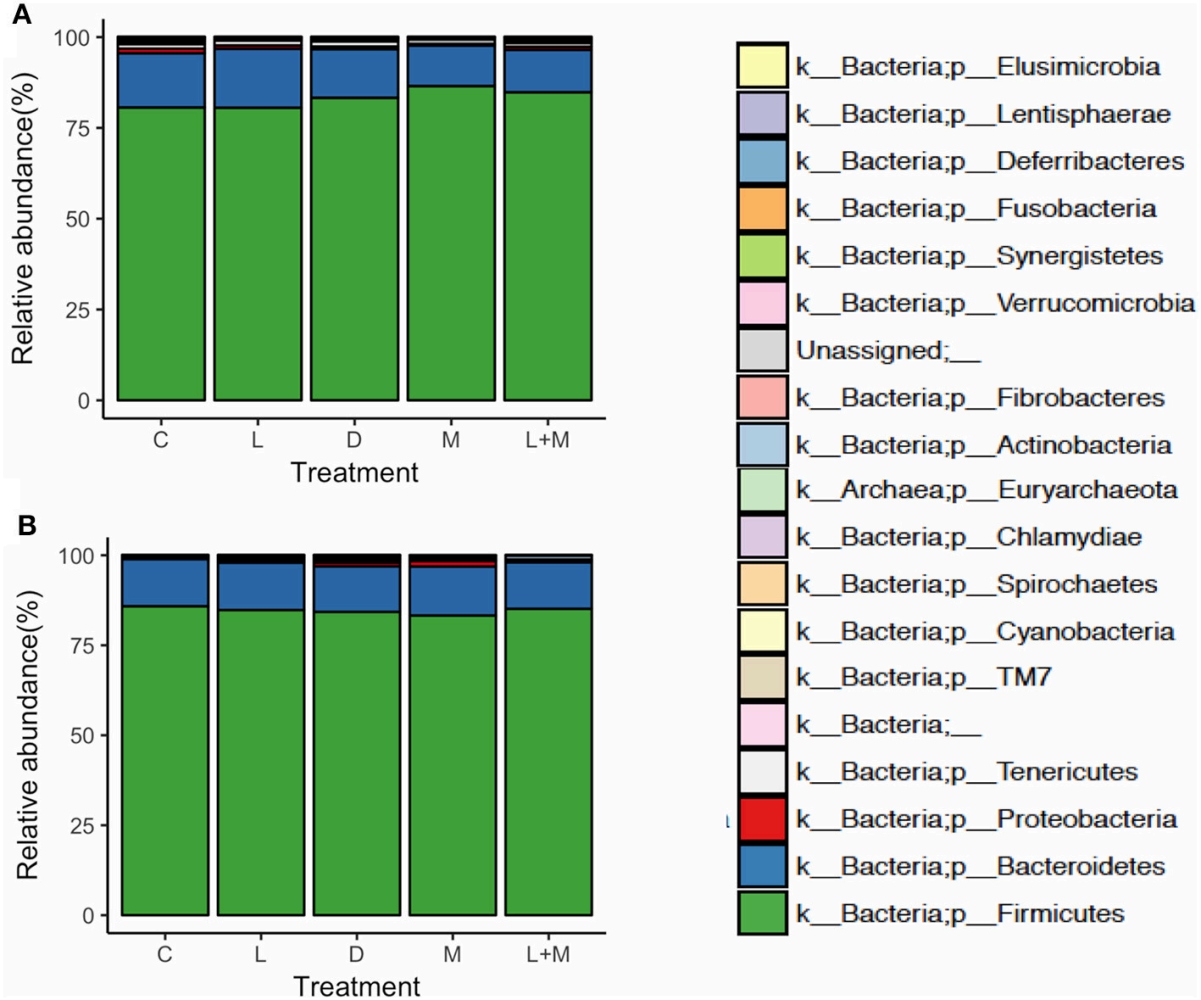
Relative abundances at the phylum level of the gut microbiota in post-weaning pigs that were fed probiotics-supplemented diets or a control diet on **(A)** the final day of Phase 1 (T2; day 40 post-weaning) and **(B)** the final day of Phase 2 (T3; day 90 post-weaning).

On T2 and T3, Firmicutes was the most dominant phylum in the gut microbiota of all treatment groups followed by Bacteroidetes (Figure 2), with these two phyla representing over 95% of the total microbiota. On T2, the proportions of five phyla (Table 5) and 11 genera (Table 6) significantly differed among treatments. At the phylum level, there was no significant difference in the relative abundance of Bacteroidetes among treatments but there was a significantly higher relative abundance of Firmicutes in M than in C, L, or D and of Cyanobacteria in C than in L, M, or L+M. At the genus level, there was a significantly higher abundance of *Lactobacillus* in C, M, and L+M than in L or D, of *Chlamydia* in C, M, and L+M than in L, and of *Treponema* in C and L+M than in L or M. On T3, the proportions of two phyla (Table 5) and eight genera (Table 6) significantly differed among treatments, with significantly higher abundances of the phylum Actinobacteria, unidentified genera in the *Coriobacteriaceae* family, and other *Coriobacteriaceae* in D, M, and L+M than in C, and of *Megasphaera* in M and L+M than in the other treatment groups.

**Table 5:** Relative abundances (%) of bacterial phyla that exhibited significant differences among treatment groups (*q* < 0.10) in the gut microbiota of post-weaning pigs.

		**Treatment**					***q*****-value**	
**Time**	**Phylum**	**C**	**L**	**D**	**M**	**L+M**	**<0.05**	**<0.10**
T2	Chlamydiae	0.20 ± 0.24	0.00 ± 0.00	0.02 ± 0.04	0.02 ± 0.02	0.13 ± 0.04	L vs. C L+M vs. L, D, M	L vs. M
	Cyanobacteria	0.24 ± 0.16	0.06 ± 0.06	0.09 ± 0.07	0.04 ± 0.01	0.04 ± 0.02	C vs. L, M, L+M	
	Firmicutes	80.6 ± 2.9	80.5 ± 5.7	83.3 ± 2.6	86.5 ± 1.2	84.8 ± 3.7		M vs. C, L, D
	Proteobacteria	1.27 ± 0.73	0.94 ± 0.29	0.76 ± 0.33	0.48 ± 0.06	0.88 ± 0.55		L vs. M
	Spirochaetes	0.22 ± 0.23	0.03 ± 0.03	0.14 ± 0.13	0.03 ± 0.05	0.15 ± 0.11		C, L+M vs. L, M
T3	Actinobacteria	0.15 ± 0.22	0.21 ± 0.34	0.75 ± 0.56	0.79 ± 0.82	1.03 ± 0.79		C, L vs. D, M, L+M
	Tenericutes	0.16 ± 0.16	0.50 ± 0.47	0.26 ± 0.30	0.011 ± 0.012	0.018 ± 0.022		L vs. M, L+M

*Values are means ± standard deviations. The significance of differences was evaluated using the Kruskal–Wallis test followed by the post hoc Mann–Whitney U-test with the Benjamin–Hochberg false discovery rate correction (q < 0.10). T2, Final day of Phase 1 (day 40 post-weaning); T3, final day of Phase 2 (day 90 post-weaning)*.

*C, Basal diet; L, basal diet + 1 g/kg of Product 1 containing live Enterococcus faecium; D, basal diet + 1 g/kg of Product 2 containing heat-killed E. faecium; M, basal diet + 1 g/kg of Product 3 containing live Clostridium butyricum; L+M, basal diet + 1 g/kg of Product 4 containing live E. faecium + C. butyricum*.

**Table 6:** Relative abundances (%) of bacterial genera that exhibited significant differences among treatment groups (*q* < 0.10) in the gut microbiota of post-weaning pigs.

			**Treatment**					***q*****-value**	
**Time**	**Phylum**	**Genus**	**C**	**L**	**D**	**M**	**L+M**	**< 0.05**	**< 0.10**
T2	Bacteroidetes	*Parabacteroides*	0.27 ± 0.16	0.12 ± 0.10	0.25 ± 0.11	0.05 ± 0.02	0.18 ± 0.14	M vs. C, D, L+M	
	Chlamydiae	*Chlamydia*	0.20 ± 0.24	0.00 ± 0.00	0.02 ± 0.04	0.02 ± 0.02	0.13 ± 0.04	L vs. C, L+M vs. L, D, M	L vs. M
	Cyanobacteria	o_YS2;f_;g_	0.24 ± 0, 16	0.06 ± 0.06	0.09 ± 0.07	0.04 ± 0.01	0.04 ± 0.02	C vs. L, M, L+M	
	Firmicutes	*Lactobacillus*	5.44 ± 2.51	1.98 ± 1.18	1.98 ± 0.99	9.18 ± 5.24	6.87 ± 4.66	L, D vs. M, L+M	C vs. L, D
	Firmicutes	*Dorea*	1.13 ± 0.25	0.61 ± 0.18	0.83 ± 0.59	1.37 ± 0.48	1.45 ± 0.73	C, M, L+M vs. L	
	Firmicutes	f_Peptostreptococcaceae;g_	0.41 ± 0.09	0.78 ± 0.21	0.54 ± 0.21	0.77 ± 0.29	0.42 ± 0.22		C, L+M vs. L, M
	Firmicutes	*Faecalibacterium*	4.01 ± 1.95	2.36 ± 0.46	2.58 ± 2.02	3.48 ± 0.95	1.66 ± 0.51	M vs. L+M	L vs. M
	Firmicutes	*Mitsuokella*	0.14 ± 0.12	0.37 ± 0.54	0.62 ± 0.81	0.59 ± 0.17	0.02 ± 0.03	C vs. M L+M vs. L, M	
	Firmicutes	*L7A_E11*	0.03 ± 0.01	0.05 ± 0.02	0.05 ± 0.06	0.02 ± 0.01	0.06 ± 0.03		L, L+M vs. M
	Proteobacteria	o_RF32;f_;g_	0.05 ± 0.04	0.006 ± 0.007	0.00 ± 0.00	0.01 ± 0.01	0.04 ± 0.05	C vs. D	C vs. L, M M vs. L+M
	Spirochaetes	*Treponema*	0.22 ± 0.23	0.03 ± 0.03	0.14 ± 0.13	0.03 ± 0.05	0.15 ± 0.11		C, L+M vs. L, M
T3	Actinobacteria	f_Coriobacteriaceae;_	0.001 ± 0.002	0.013 ± 0.031	0.06 ± 0.40	0.038 ± 0.022	0.083 ± 0.045	C vs. D, M, L+M L vs. D, L+M	
	Actinobacteria	f_Coriobacteriaceae;g_	0.036 ± 0.025	0.048 ± 0.080	0.17 ± 0.10	0.22 ± 0.18	0.36 ± 0.29	C vs. D, M, L+M L vs. L+M	L vs. D
	Actinobacteria	*Collinsella*	0.013 ± 0.014	0.013 ± 0.033	0.087 ± 0.068	0.053 ± 0.027	0.16 ± 0.15	C, L vs. D, M, L+M M vs. L+M	
	Bacteroidetes	f_S24-7;g_	0.25 ± 0.16	0.50 ± 0.31	0.90 ± 0.76	0.86 ± 0.90	1.20 ± 0.52		C vs. L+M
	Firmicutes	*Megasphaera*	5.95 ± 1.29	4.04 ± 4.28	7.21 ± 2.02	13.46 ± 2.84	12.55 ± 3.75	C, L, D vs. M, L+M	
	Firmicutes	*Mogibacterium*	0.052 ± 0.040	0.069 ± 0.051	0.011 ± 0.010	0.003 ± 0.008	0.012 ± 0.020	L vs. M	C vs. M L vs. D, L+M
	Proteobacteria	*Escherichia*	0.028 ± 0.063	0.003 ± 0.006	0.068 ± 0.080	0.89 ± 2.17	0.076 ± 0.17		L vs. D
	Tenericutes	o_RF39;f_;g_	0.16 ± 0.15	0.48 ± 0.47	0.24 ± 0.28	0.011 ± 0.012	0.018 ± 0.021		L vs. M, L+M

*Values are means ± standard deviations. “o,” “f,” and “g” indicate the order, family, and genus, respectively. Some operational taxonomic units (OTUs) could not be assigned to a genus because the sequence was absent from the database. The significance of differences was evaluated using the Kruskal–Wallis test followed by the post hoc Mann–Whitney U-test with the Benjamin–Hochberg false discovery rate correction (q < 0.10). T2, Final day of Phase 1 (day 40 post-weaning); T3, final day of Phase 2 (day 90 post-weaning)*.

## Discussion

In this study, we examined the effects of dietary supplementation with *E. faecium* and *C. butyricum*, both individually and in combination, on the growth and gut microbiota composition of post-weaning pigs. We found that the addition of live or heat-killed *E. faecium* to the pigs' diet during the post-weaning period improved their growth performance, with no significant difference between the two treatments. Similarly, Sukegawa et al. (8) reported that dietary supplementation with heat-killed *E. faecium* enhanced the growth of post-weaning pigs. Heat-killed bacterium cannot produce any metabolites. The bacterial components might have positive effect on the gut, resulting in increasing BW. On the other hand, supplementation with *C. butyricum* did not have any effect on growth performance in the present study. This contrasts with the findings of Takahashi et al. (5), who reported that the use of *C. butyricum* may improve the zootechnical performance of weaned piglets. The effect of dietary probiotics varies depending on factors such as location and the microbiota of the host. Moreover, in the present study, the amount of feeding *C. butyricum* was 9.6 × 10^4^ cfu/g feed, which was less than that of the previous study. For these reasons, the BW of pigs fed *C. butyricum* might have not increased contrast to that of control treatment. Following completion of the probiotics administration period, there was no significant difference in the BW of pigs among treatments, indicating that there was no residual effect of the probiotics on growth performance. Beneficial effects of these probiotics might be diminished by ceasing probiotics, resulting in the growth performance of pigs in probiotic treatments returning back to normal. The result implies that the bacteria were not able to persist in the intestine.

We expected that the combined use of *E. faecium* and *C. butyricum* would have a highly synergistic effect on the pigs due to the different characteristics of LAB and BAB. However, we found that pigs in the L+M treatment group had a similar BW to those in the L and M groups, indicating that the use of single probiotics had a similar effect on growth performance to the combined use of *E. faecium* and *C. butyricum*. Similarly, Han et al. (17) reported that the combined supplementation of broilers with *C. butyricum* and *L. plantarum* had no significant effect on growth performance. Many studies have demonstrated that dietary LAB and BAB have positive effects on factors other than growth parameters in animals, such as intestinal morphometric parameters. For example, the length of the intestinal villi is improved by feeding pigs LAB (18–20) and broilers BAB (21). Moreover, Long et al. (22) reported that the combined use of LAB and BAB could further improve the length of the intestinal villi in mice. Therefore, since we were unable to investigate factors associated with intestinal health in the present study, we cannot rule out the possibility that the combined use of LAB and BAB has synergistic effects on factors other than growth parameters and the microbiota of post-weaning pigs.

We also explored the effects of dietary supplemented probiotics on the diversity of the gut microbiota in post-weaning pigs, using Chao1 to indicate the bacterial richness and the Shannon index to reflect the bacterial diversity. We found no significant differences in alpha diversity among treatments. Previous studies have obtained varying results on the effects of probiotics on alpha diversity–for example, Chae et al. (23) showed that the administration of *E. faecium* to weaned piglets increased the bacterial richness, while Li et al. (24) showed that dietary supplementation with *E. faecalis* decreased the bacterial richness. Thus, the effects of probiotics on the alpha diversity of the gut microbiota appears to vary depending on the microorganism strain, diet, and environmental factors.

To confirm this lack of alteration of the intestinal microbiota by these dietary probiotics, we performed PCoA based on weighted UniFrac distances. Interestingly, this showed that there were significant changes in microbial community structure following the administration of *E. faecium* and *C. butyricum*. Furthermore, significant differences in beta diversity were still observed between M and C following completion of the probiotics treatment period, indicating that the effect of dietary *C. butyricum* on the structure of the microbiota was sustained.

Many researchers have reported that Firmicutes and Bacteroidetes are the most dominant phyla in pig fecal samples (23, 25–27) and our findings supported this. Bacteria in the phylum Firmicutes are fiber digesters and produce short-chain fatty acids (SCFA) from dietary compounds (28), resulting in improved growth performance. Although there was no significant difference in the BW of pigs between M and the other treatment groups, supplementation with only *C. butyricum* increased the proportion of Firmicutes on T2. Bacteria in the genus *Lactobacillus* (phylum Firmicutes) are beneficial in the intestine, producing bacteriocins, organic acids, and hydrogen peroxide (29), and many studies have shown that dietary probiotics increase the relative abundance of *Lactobacillus* in pigs (23, 30, 31). Furthermore, we previously found that dietary supplementation with heat-killed *E. faecium* increased the proportion of *Lactobacillus* (8). However, in the present study, the relative abundance of *Lactobacillus* was significantly lower in pigs that had been supplemented with live or heat-killed *E. faecium* than in the control, and in a separate study conducted at another commercial farm, we found that pigs had similar proportions of *Lactobacillus* in their gut microbiota regardless of whether their diets were supplemented with live, heat-killed, or no *E. faecium* (data not shown). Therefore, it appears that the effect of live and heat-killed *E. faecium* on *Lactobacillus* may vary depending on the microbiota of the host and the dietary probiotic used, and it is not necessarily related to a reduction in *Lactobacillus*.

Bacteria in the phylum Chlamydiae are associated with a broad range of diseases in swine and Pollmann et al. (32) reported that dietary *E. faecium* reduced the incidence of *Chlamydia* infection. Similarly, in the present study, we found that dietary supplementation with live *E. faecium* decreased the proportion of *Chlamydia*. Furthermore, the administration of live *E. faecium* and *C. butyricum* decreased the proportion of *Treponema* (phylum Spirochaetes), suggesting that these probiotics inhibited the growth of these bacteria, which are associated with colitis (33).

Dietary supplementation with *C. butyricum* has also been shown to increase the relative abundance of *Megasphaera* (phylum Firmicutes) (34), which includes *Megasphaera elsdenii*, an intestinal lactate- and sugar-fermenting species (35) that produces SCFAs, which are important for the energy balance of animals (27). Similarly, we found that the proportion of *Megasphaera* in the gut microbiota was significantly higher in the M and L+M treatment groups than in C on T3. We did not, however, observe any significant difference among treatments on T2, the reason for which is unclear but may be related to dietary *C. butyricum* affecting the growth of *Megasphaera*. After completion of the probiotics treatment period, there was no significant difference in the relative abundance of any taxonomic group except Cyanobacteria between L+M and C, suggesting that combined administration of *E. faecium* and *C. butyricum* did not have a synergistic effect on the composition of the gut microbiota.

In conclusion, we demonstrated that dietary supplementation with live or heat-killed *E. faecium* during the post-weaning period enhanced the growth performance of pigs and that the use of *E. faecium* or *C. butyricum* altered the structure of the microbiota. However, we did not observe any synergistic effect of their combined use on growth performance and taxonomic composition. Further studies are required to evaluate the combined effects of these probiotics on other factors, such as the intestinal health of pigs.

## Author Contributions

YS performed the experiments, analyzed the data, and wrote the manuscript. YK and SR analyzed the data. KO, MT, and TF designed the study. SS designed the study and analyzed the data. All authors discussed the results and approved the final manuscript.

### Conflict of Interest Statement

YS, SR, SS, and TF were employed by NH Foods Ltd. YK, KO, and MT were employed by Miyarisan Pharmaceutical Co., Ltd.
